# SHP-2 Expression Is a Positive Prognostic Biomarker in Non-Small-Cell Lung Carcinoma: Association with KRAS Mutation and Prolonged Survival

**DOI:** 10.3390/pathophysiology33030052

**Published:** 2026-07-18

**Authors:** Konstantinos Stamopoulos, Georgios Pilichos, Eleni Karatrasoglou, Penelope Korkolopoulou, Stratigoula Sakellariou

**Affiliations:** 1 Second Department of Internal Medicine, General and Oncology Hospital of Kifissia “Agioi Anargyroi”, Kifissia, 13561 Athens, Greece; k_stamopoulos@hotmail.com; 2 Thoracic Surgery Department, Sotiria General Hospital for Chest Diseases, 11527 Athens, Greece; giwrgospilihos@gmail.com; 3 First Department of Pathology, Laiko General Hospital, Medical School, National and Kapodistrian University of Athens, 11527 Athens, Greece; elina_karat@hotmail.com (E.K.); pkorkol@med.uoa.gr (P.K.)

**Keywords:** non-small cell lung cancer, SHP-2, PTPN11, adenocarcinoma, squamous cell carcinoma, immunotherapy, PD-L1

## Abstract

**Background/Objectives**: Lung cancer tumors are the most frequent malignant tumors and the primary cause of cancer-related deaths. Despite the improvement in survival related to the implementation of immunotherapy, a significant proportion of patients fail to demonstrate satisfactory response. As a result, the need for new biomarkers, which will predict patient response, as well as for novel therapeutic targets, is urgent. SHP-2 is an intracellular signal transduction molecule, modifying signal transduction of numerous intracellular cascades, acting primarily as an oncogene. In this study we analyzed the expression pattern of SHP-2 in a cohort of non-small-cell lung cancer cases and attempted to correlate it with available clinicopathological parameters. **Methods**: We performed immunohistochemistry for SHP-2 detection in a cohort of 258 NSCLC cases. H-score was estimated and statistical analyses correlated it with survival and other clinicopathological and molecular parameters. **Results**: SHP-2 expression was observed in 60 cases (23.3%), with an H-score ranging from 150 to 300. Positive samples were distributed among the three major histological subtypes, without statistically significant differences. Statistical analysis revealed significant positive correlation of SHP-2 expression with smoking, PD-L1 levels, and *KRAS* mutations. Moreover, positive SHP-2 immunohistochemistry was associated with better clinical outcome in both the total cohort and in the group of patients who received immunotherapy. Finally, the simultaneous presence of SHP-2 expression and *KRAS* mutation was correlated with prolonged survival. **Conclusions**: Our findings indicate that SHP-2 could be a useful prognostic factor and a very reliable biomarker in predicting response to immune checkpoint inhibitors.

## 1. Introduction

Lung cancer is the most common malignancy and the most frequent cause of cancer-related deaths on a global scale, equally affecting men and women and showing a strong correlation with smoking [1,2,3]. It is characterized by high histological and molecular heterogeneity. From a histological perspective, it has been classically segregated into small-cell and non-small-cell lung cancer (SCLC and NSCLC), with the former category representing aggressive tumors with neuroendocrine differentiation and the latter encompassing both adeno- and squamous cell malignancies. NSCLC accounts for the vast majority of disease cases [4,5,6]. Squamous cell tumors arise characteristically in large airways, originating from squamous metaplasia of the bronchial epithelium, and are primarily driven by *TP53* inactivating mutations [7,8]. On the other hand, the majority of adenocarcinomas occur in peripheral locations, with type II pneumocytes considered as their cell of origin, and display much higher histological and molecular variability [9]. While *EGFR* and *KRAS* gain-of-function mutations represent the most prevalent genetic alterations, additional genes have been identified as major factors in disease pathogenesis, such as *ALK*, *ROS1*, and *NTRK* [10,11]. In general, *KRAS*-activating somatic alterations are associated with dismal prognosis and worse PFS and OS, as observed in a meta-analysis of 43 studies [12]. On the other hand, *ALK* translocations correlate with better PFS and OS compared to those lacking *ALK* genetic alterations, underlying the role of ALK as a favorable prognostic factor [13]. Besides offering a deeper insight into the biology, these genomic alterations have a profound impact on the treatment of lung carcinomas as they generate actionable molecules, which serve as targets for therapeutic intervention.

Lung cancer is one of the first malignancies where immune checkpoint inhibitors (ICIs), especially anti-PD-1/anti PD-L1 and anti-CTLA-4 agents, were implemented with promising results. These new therapeutic approaches have been incorporated into everyday clinical practice and are usually used in combination with traditional surgical, chemotherapeutic, and radiotherapy treatments [14,15]. Despite the promising results observed in a proportion of cases, the majority of lung carcinoma patients fail to demonstrate satisfactory response [16,17]. As a result, there is an urgent need for the establishment of standardized criteria that will allow the selection of patients who are more likely to experience substantial survival benefit from immunotherapy. The expression levels of PD-L1 by both neoplastic and immune cells is, at the moment, the only biomarker recommended by the National Comprehensive Cancer Network (NCCN) and regularly used to determine patient suitability [18,19,20]. However, experience so far has proved that the presence of PD-L1 is a necessary but not sufficient condition for the successful implementation of immune checkpoint blockade, as a lot of factors interfere with the interaction of neoplastic cells with the host immune system and consequently affect the development of immune response. Tumor mutational burden (TMB), reflecting the abundance of cancer cells, neoantigens, and genetic alterations with a direct impact on the recruitment and activation of immune cells in the tumor microenvironment, are two additional parameters associated with the response to ICIs [21]. An extensive meta-analysis conducted on 28 studies, including a total of about 3500 patients, compared the efficacy of PD-1/PD-L1 treatment either as monotherapy or combined with anti-CTLA4 agents with chemotherapy in cases of low or high TMB. As expected, ICIs were associated with prolonged OS and PFS in patients with high TMB, while in the low TMB group, chemotherapy was associated with better PFS and OS did not show a significant difference between the two treatment approaches [22]. Despite the robust findings supporting the predictive role of TMB in immunotherapy response, it has not been incorporated in routine clinical practice yet. A better understanding of tumor–immune system interactions is therefore required so that reliable markers of ICI efficacy can be identified. In this setting, future research efforts should aim to decipher the exact mechanisms that tumor cells implement in order to avoid immune surveillance.

SHP-2 is an intracellular signal transduction molecule encoded by the *PTPN11* gene. It serves as a tyrosine phosphatase and contains a catalytic PTP domain, which exerts phosphate group cleavage activity, and two SH2 domains, which controls its interactions with other cellular proteins. SHP-2 is recruited by multiple tyrosine kinase receptors (RTKs) and modifies the signal transduction of numerous intracellular cascades, acting primarily as an oncogene [23,24]. While its most well-characterized partner is RAS and the most thoroughly studied pathway it interferes with is RAS-RAF-MEK-ERK, it has been found to enhance the downstream signaling of PI3K/Akt and JAK/STAT as well, in a tumor-promoting manner [25,26,27,28]. It also plays a crucial role in immune response by positively modulating PD-1 signaling, leading to attenuation of the CD3/ZAP70 cascade, and consequently facilitating suppression of adaptive immune cell activation. This pivotal immunomodulatory role of SHP-2 renders it a promising target, which could be utilized in combination treatment schemes, along with ICIs, with the potential to boost immunotherapy efficacy [29,30].

In this study, we analyzed the expression pattern of SHP-2 in a cohort of NSCLC specimens and correlated it with several clinicopathological parameters, including patient survival, providing innovative data on its potential role as a biomarker and therapeutic target.

## 2. Materials and Methods

### 2.1. Study Cohort

The present study was carried out in the First Department of Pathology, National and Kapodistrian University of Athens School of Medicine, Laiko General Hospital (NKUA), and was approved by the Ethics Committee of NKUA (Protocol No. 1718004921). Our cohort consisted of 258 patients, for whom formalin-fixed paraffin-embedded (FFPE) tissues were available in the archive of the department, derived primarily from biopsies or fine needle aspirates from thoracic sites (lung, lymph nodes, pleura), as well as from surgical specimens. A small number of samples consisted of brain or bone metastasis biopsies. All patients included in our study had a histologically confirmed diagnosis of NSCLC, which had been established based on criteria set by the Tumor Histology International Association for the study of Lung Cancer/American Thoracic Society/European Respiratory Society International Multidisciplinary Classification of Lung Adenocarcinoma/2011, WHO 5th edition 2021 [31].

Demographic, clinical, and pathologic characteristics were also available, including age, gender, history of smoking, tumor histology, administration of immunotherapy, progression-free-survival (PFS), and overall survival (OS). Follow-up data were collected via clinical and radiological evaluation every 3 to 6 months, according to institutional protocols. For both PFS and OS estimation, treatment initiation was used as a starting point. PFS was defined as the period until documented disease progression, while OS was calculated until the date of death or last follow-up.

Moreover, numerous immunohistochemical and molecular analyses had taken place in our cohort samples, including immunohistochemistry for PD-L1, immunohistochemistry and FISH for ALK, immunohistochemistry and CISH for ROS1, as well as genome sequencing for the detection of *KRAS*, *EGFR,* and *BRAF* mutations. For a detailed analysis of the protocols implemented for these analyses, please look at our previous publication [32].

### 2.2. SHP-2 Immunohistochemistry

SHP-2 immunohistochemistry was performed on 4 μm thick FFPE sections, using the SHP-2 (D50F2) rabbit monoclonal antibody from Cell Signaling Technology. Staining was evaluated by two experienced pathologists (PK and SS) and the H-score was estimated for each sample, in a range of 0–300, by multiplying the percentage of positive cells (0–100) with the intensity of staining (0: no staining, 1: mild intensity, 2: moderate intensity, 3: strong intensity) [33]. Based on a previous paper, we considered as positive the samples with a H-score over 150 [34].

### 2.3. Statistical Analysis

Statistical analysis was performed in order to correlate SHP-2 expression with the available clinicopathological, immunohistochemical, and molecular data, using Pearson’s chi-square test with continuity correction or Fisher’s exact test, as appropriate. All tests were two-sided, and the level of statistical significance was set at 0.05. Differences in progressive-free survival (PFS) and overall survival (OS) between groups were estimated by the log-rank test and represented by Kaplan–Meier curves.

## 3. Results

### 3.1. Epidemiological and Clinical Characteristics

Our cohort consisted of 258 patients, including 184 (71.3%) men and 74 (28.7%) women, with a median age of 66 years old (range: 34–88). The majority of them, namely 200 patients (77.5%), had a history of smoking. Regarding the histological profile of tumors, adenocarcinoma was the most common subtype, accounting for 219 cases (84.9%), with squamous cell carcinoma coming second with 28 cases (10.9%). Ten patients’ neoplasms (3.9%) had been identified as NSCLC not otherwise specified (NSCLC-NOS), while one case of a well-differentiated neuroendocrine tumor (lung carcinoid) completed the study population. Concerning the clinical outcome of our cases, their median OS was 11 months (range: 1–50), while their PFS was just 3 months (range: 0–36). Immunotherapy in the form of immune checkpoint inhibitors was included in the therapeutic protocol of 136 patients (52.7%). The main molecules utilized were inhibitors of PD-1/PD-L1. Table 1 summarizes epidemiological and clinical features of our cohort.

### 3.2. Molecular and Immunohistochemical Characteristics

As already mentioned, sequencing analyses for the identification of *EGFR*, *KRAS,* and *BRAF* mutations, in situ hybridization for the detection of *ALK* amplification, and immunohistochemical assessment of ROS1 and PD-L1 expression were also available.

All *EGFR*, *KRAS*, and *BRAF* mutations were encountered in adenocarcinomas (Table 2). The most commonly mutated gene was *KRAS*, with gain-of-function alterations identified in 70 specimens (27.1%), followed by *EGFR*, which displayed activating mutations in 24 cases (9.3%). The most prevalent *EGFR* genetic perturbation was exon 19 deletion (Ex19del), detected in 15 cases (5.8%), followed by exon 20 insertion (Ex20ins) and exon 21 point-mutation (L858R) observed in five (1.9%) and four (1.6%) tumors, respectively. BRAF changes were encountered in only five adenocarcinomas of our cohort (1.9%), while amplification of the *ALK* locus was detected in nine lung adenocarcinomas (3.5%).

**Figure 1:**
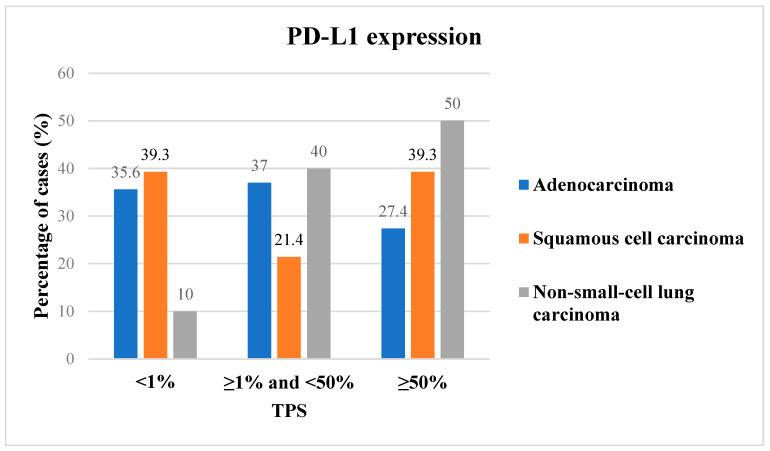
PD-L1 expression levels among the three different histological subtypes of lung tumors.

Concerning immunohistochemical markers, ROS1 expression was observed in 21 specimens (8.1%) with a H-score ranging from 120 to 250. Twenty of the 21 positive tumors were adenocarcinomas, and one of them was a NSCLC-NOS. PD-L1 expression was observed in 189 samples, with the median percentage of positive cancer cells (TPS) standing at 5% (range: 0–95%). The staining levels of PD-L1 demonstrated significant variation, with 90 specimens (34.9%) showing a TPS of less than 1%, 92 lying between 1% and 50% (35.7%), and the remaining 76 cases (29.4%) displaying >50% positive tumor cells (Figure 1). In contrast to ROS1 expression, PD-L1 was more homogeneously distributed among different histological subtypes, with positive staining observed in 160/219 (73.1%) adenocarcinomas, 19/28 (67.9%) squamous cell carcinomas, and 9/10 (90%) NSCLC-NOS.

### 3.3. SHP-2 Expression and Statistical Correlations with Other Clinicopathological Parameters

**Figure 2:**
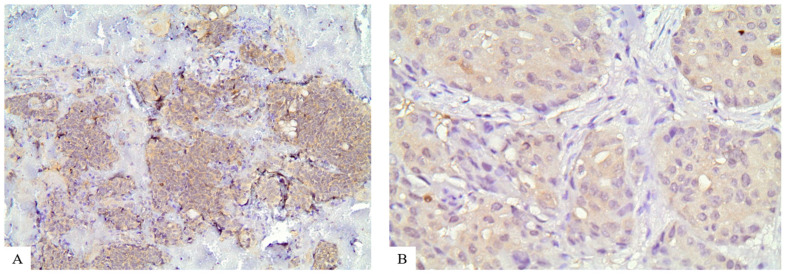
Cytoplasmic expression of SHP-2 in a squamous cell lung carcinoma: (**A**) ×200; (**B**) ×400.

**Figure 3:**
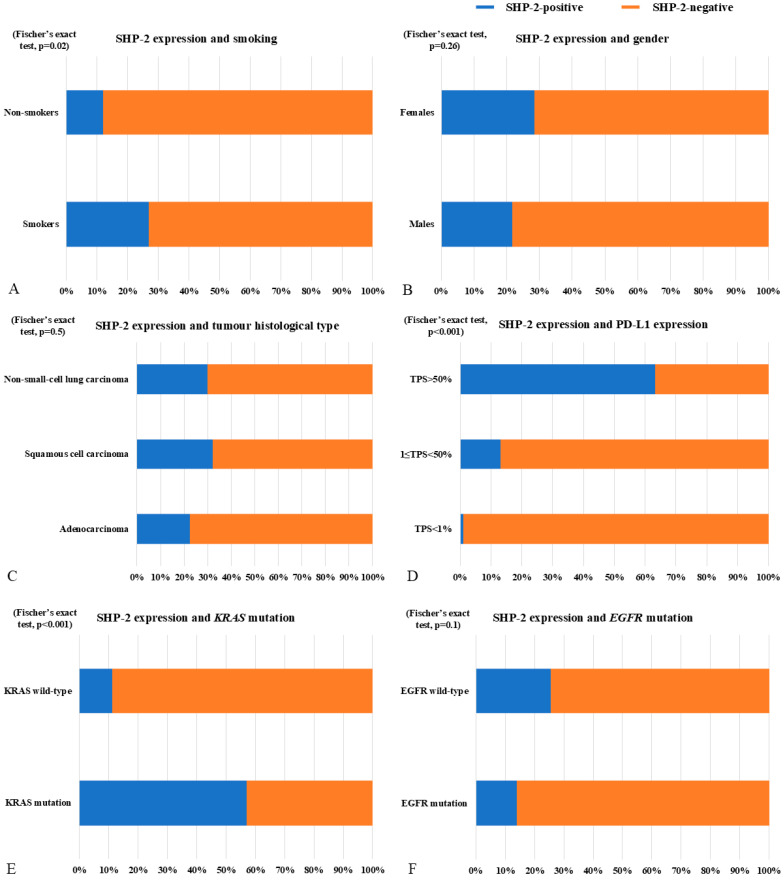
Correlation of SHP-2 expression with multiple clinicopathological and molecular parameters: (**A**) smoking (Fisher’s exact test, *p* = 0.02), (**B**) gender (Fisher’s exact test, *p* = 0.26, (**C**) tumor histological type (Fisher’s exact test, *p* = 0.5), (**D**) PD-L1 expression (Fisher’s exact test, *p* < 0.001), (**E**) *KRAS* mutation (Fisher’s exact test, *p* < 0.001), and (**F**) *EGFR* mutation (Fisher’s exact test, *p* = 0.1).

SHP-2 staining was considered positive in 60 samples (23.3%), with exclusively cytoplasmic localization (Figure 2), and distributed among the three major histological subtypes without statistically significant differences (Fisher’s exact test, *p* = 0.5) (Figure 3). The H-score of positive cases ranged between 150 and 300. While adenocarcinomas accounted for the majority of positive specimens, namely 49 (22.4%), the highest positivity frequency was detected in tumors of squamous differentiation, with nine of them (32.1%) displaying SHP-2 expression. The two remaining positive neoplasms were NSCLC-NOS (20%) (Table 3).

Additional statistical analyses revealed a very strong correlation of SHP-2 staining with PD-L1 expression levels (Fisher’s exact test, *p* < 0.001) and with the presence of *KRAS* mutations (Fisher’s exact test, *p* < 0.001) (Figure 3). More specifically, moving from low (<1%) to moderate (≥1%, <50%) and to high (≥50%) PD-L1 expression, the frequency of SHP-2 positivity progressively increased, rising from 1/90 (1.1%) to 12/92 (13%) and reaching a peak of 48/76 (43.2%) in the high PD-L1 expressors group (Figure 3). Regarding *KRAS* genetic alterations, they were detected in 40/70 (57.1%) SHP-2-positive cases compared to 21/189 (11.2%) SHP-2-negative cases. A statistically significant association was also identified between SHP-2 expression and smoking status (Fisher’s exact test, *p* = 0.02), as a higher SHP-2 positivity frequency was encountered in smokers (40/184, 21.7%) compared to non-smokers (7/58, 12.1%) (Figure 3). No statistically significant correlation was observed between SHP-2 staining and gender, *EGFR* mutational status, as well as with ALK and ROS1 expression (Fisher’s exact test, *p* > 0.05) (Figure 3).

### 3.4. Survival Analyses

**Figure 4:**
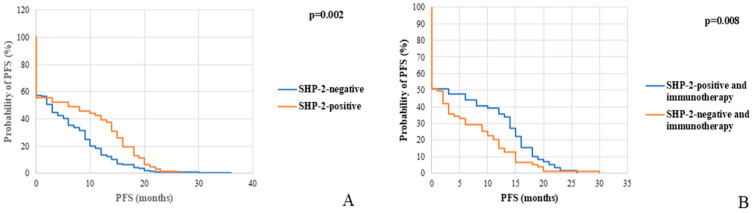
Kaplan–Meier curves correlating SHP-2 expression with survival in the whole cohort ((**A**), log-rank test, *p* = 0.002) and among the patients who received immunotherapy ((**B**), log-rank test, *p* = 0.008).

Expression levels of SHP-2 showcased a statistically significant correlation with clinical outcomes (OS and PFS). Positive SHP-2 immunohistochemical staining was associated with prolonged PFS (log-rank test, *p* = 0.002) in the total cohort (Figure 4). A mildly weaker association (log-rank test, *p* = 0.008) was observed among patients who received immunotherapy, as cases with SHP-2 expression showcased longer PFS compared to those lacking SHP-2 (Figure 4).

**Figure 5:**
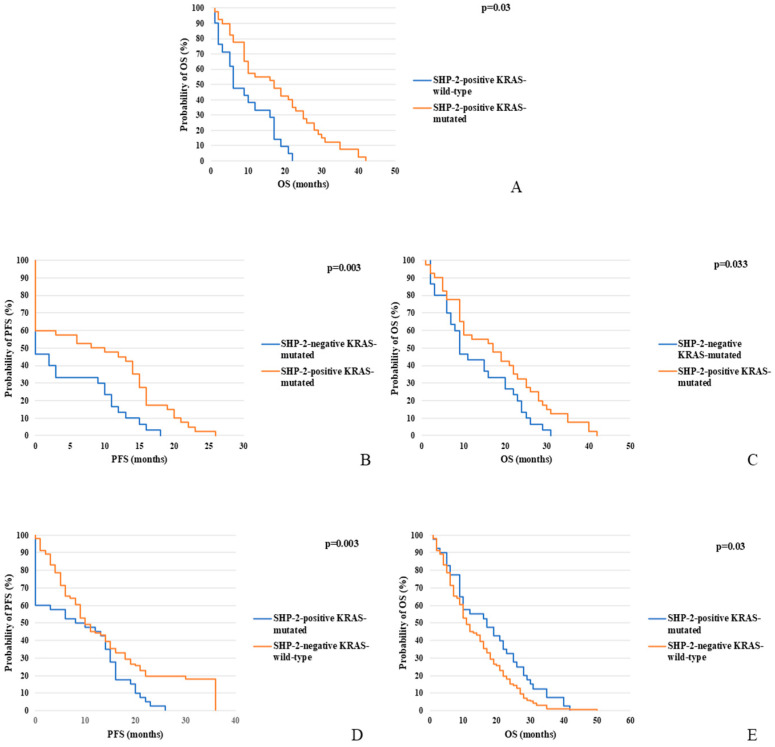
Kaplan–Meier curves correlating SHP-2 expression and *KRAS* mutational status with survival. (**A**) Correlation of *KRAS* mutation with OS in SHP-2-positive cases (log-rank test, *p* = 0.03). (**B**,**C**) Correlation of SHP-2 expression with PFS (log-rank test, *p* = 0.03) and OS (log-rank test, *p* = 0.033) among *KRAS*-mutant cases. (**D**,**E**) Correlation of simultaneous SHP-2 expression and *KRAS* mutation with PFS (log-rank test, *p* = 0.003) and OS (log-rank test, *p* = 0.03).

Interesting findings were also extracted from the multiple different combinations of SHP-2 expression and *KRAS* mutational status (Figure 5). First of all, in SHP-2-positive cases, the presence of *KRAS* gain-of-function mutations was correlated with prolonged OS (log-rank test, *p* = 0.03). On the other hand, in patients with *KRAS*-mutant neoplasms, SHP-2 expression was correlated with improved PFS (log-rank test, *p* = 0.003) and OS (log-rank test, *p* = 0.033). Finally, the concurrent presence of SHP-2 immunohistochemical staining and *KRAS* mutation was also associated with better PFS (log-rank test, *p* = 0.003) and OS (log-rank test, *p* = 0.03).

## 4. Discussion

In this study we analyzed the expression pattern of SHP-2 using immunohistochemistry in a large cohort of NSCLC cases and attempted to correlate it with available clinicopathological and molecular data.

This is, to our knowledge, the first comprehensive review of SHP-2 expression in such an extensive pool of NSCLCs. Overall, SHP-2 staining was considered positive in 60 of the 258 samples (23.3%) and showed statistically significant correlation with smoking; the positive cases were distributed among different histological subtypes, without statistically significant variations. More specifically, SHP-2 expression was observed in 49/219 (22.4%) adenocarcinomas, 9/29 (32.1%) squamous cell carcinomas, and 2/10 (20%) NSCLCs. SHP-2 levels were also positively correlated with smoking, the presence of *KRAS* mutation, and PD-L1 expression. Another interesting finding concerns the role of SHP-2 as a prognostic and predictive marker capable of estimating a patient’s survival and response to immunotherapy. In more detail, SHP-2 expression was associated with prolonged PFS in both the total patient cohort (log-rank test, *p* = 0.002) and specifically in those who received immunotherapy with checkpoint inhibitors (log-rank test, *p* = 0.008). Especially in the group of patients who received immunotherapy schemes, the most straightforward explanation could be the increased levels of PD-L1 encountered in SHP-2-positive specimens. Significant correlations regarding patient survival were also identified in relation to *KRAS* mutational status. In general, the co-existence of SHP-2 expression and *KRAS* gain-of-function mutation was associated with better PFS and OS, regardless of the treatment protocol implemented.

A number of other studies have analyzed the role of SHP-2 in lung tumors via multiple methods, from protein expression evaluation by immunohistochemistry to molecular detection and experimental approaches. One of them utilized a tissue microarray of 80 NSCLC cases to assess SHP-2 expression and revealed increased immunohistochemical staining in neoplastic compared to tumor-adjacent tissue. Moreover, a statistically significant correlation was observed between SHP-2 expression levels and the presence of lymph node metastases [35]. Another study analyzed the expression pattern of *KRAS* and *PTPN11* genes via The Cancer Genome Atlas (TCGA) database and revealed a very strong positive statistical correlation, while SHP-2 levels were lower in *KRAS*-wild-type compared to *KRAS*-mutant samples. In addition, lower SHP-2 was associated with improved prognosis and better overall survival [36]. In addition to the assessment of SHP-2 expression levels, mutations in different foci of the gene locus have been identified. Bentires Alj et al. analyzed 65 different NSCLC cell lines and found two distinct alterations in the *PTPN11* sequence (V45L, N85S) [37].

Despite the limited and preliminary data on the role of SHP-2 in lung carcinomas, a number of studies have already evaluated its potential as a therapeutic target via both in vitro and in vivo experiments. A genetically engineered mouse model evaluated the oncogenic capacity of SHP-2 via a doxycycline (Dox)-inducible gain-of-function SHP-2 mutation, activated exclusively in Clara cells, by a Clara cell secretory protein (CCSP)-reverse tetracycline transactivator (rtTA). Mice developed both bronchiolar adenomas and lung adenocarcinomas, which regressed after Dox withdrawal. Moreover, all lung tumors, both benign and malignant, demonstrated strong pERK1/2 immunohistochemical staining, implying a pivotal role of the MAPK signaling cascade in SHP-2 tumorigenic function [38]. Another study tested the effect of a SHP-2 inhibitor, termed embelin, in *KRAS*-driven lung adenocarcinoma cell lines. Embelin is a naturally occurring para-benzoquinone, blocking the SHP-2 catalytic domain. Indeed, it was found to reduce proliferation and induce apoptosis of NSCLC cells. It also suppressed their migratory capacity and invasive potential, an effect that was accompanied by a downregulation in epithelial-to-mesenchymal transition (EMT) factors. This multidimensional anti-tumor activity was also verified in lung cancer heterotopic xenograft models [36]. Another natural compound which has displayed a tumoricidal effect against *KRAS*-mutant NSCLC cell lines is 3-acetoxylteuvincenone G (3-AG), which is an abietane diterpenoid extracted from the whole plants of Ajuga ovalifolia var. calantha. It has been shown to bind to the catalytic domain of SHP-2, blocking its phosphorylation capacity, leading to the suppression of cell proliferation and invasion and induction of A549 cell line apoptosis [39]. Another SHP-2 inhibitor showed little effect when tested on its own in a lung cancer cell line, while it significantly amplified the cytotoxic effect of MEK inhibitors when evaluated in combination schemes. This finding was also confirmed genetically: the CRISPR-associated protein 9 (Cas9)-mediated deletion of *PTPN11* increased sensitivity to MEK blockade [40]. In contrast to the above findings, in vivo experiments showcased a robust anti-tumor effect of *PTPN11* knock-out even in the absence of MEK inhibition [40]. SHP-2 blockade is also a promising approach in overcoming resistance to tyrosine kinase inhibition. TNO55 is a SHP-2-targeting molecule which has been evaluated in the setting of combination schemes, along with EGFR and KRAS inhibitors in lung cancer cell lines, preventing the emergence of acquired resistance and enhancing their tumoricidal effect [41,42]. Another interesting study conducted in broadly used lung adenocarcinoma cell lines revealed that ERK phosphorylation upon EGF and TNFa stimulation was suppressed in the presence of allosteric SHP-2 inhibitors SHP099 and TNO155. SHP-2 blockade also lead to a similar inhibitory effect on TNFa-induced EphA2 activation, which was also accompanied by diminished mobility and invasive potential of cells in wound-healing assays [43].

Besides the direct effect on cancer cells, SHP-2 is also crucial in the crosstalk between the tumor and immune microenvironments, rendering its targeting as a promising strategy in improving the response to immunotherapy with checkpoint inhibitors. Experiments in an *EGFR*-mutant lung adenocarcinoma cell line identified SHP-2 as a key driver of interferon-γ (IFN-γ) signaling suppression in tumor cells. *EGFR* gain-of-function mutations led to increased levels of IFN receptors (IFNG1, IFNG2), which was paradoxically accompanied by blunted pathway activation. The alleviation of IFN-γ signaling was associated with increased STAT1 de-phosphorylation, which was mediated by SHP-2, as the genetic deletion of *PTPN11* in the same cell line reversed this effect. In further support of this finding, combination of a PD-1 inhibitor and a SHP-2-targeting molecule led to a synergistic therapeutic effect in a heterotopic xenograft mouse model of lung adenocarcinoma, implying a role in increasing tumor responsiveness to immune checkpoint inhibitors (ICBs) [44]. In contrast to these results, and in agreement with our findings, a study correlating SHP-2 expression patterns with response to ICIs in a cohort of NSCLC cases revealed that higher SHP-2 levels were associated with better response in *KRAS*-mutant neoplasms [45]. It is therefore crucial to take into consideration the context, especially the genetic background of tumors, in order to predict the possible effect of SHP-2 in immune response and immunotherapy efficacy. It is also important to always consider the limitations of each method, both immunohistochemical and in vivo/in vitro experimental studies.

Interesting findings of distinct SHP-2 levels in tumor cells compared to non-neoplastic tissues have also been described in a variety of other solid malignancies of heterogeneous histogenesis [46]. The most characteristic examples include neoplasms of the gastrointestinal tract as well as of pancreatic origin. In pancreatic and gastric cancer, a similar pattern to NSCLCs of increased SHP-2 expression in neoplastic cells, compared to non-neoplastic cells, was encountered [47,48]. On the contrary, a downregulation of SHP-2 was detected in esophageal squamous cell carcinomas in comparison to normal tissue [49]. Studies on colorectal lesions in both pre-invasive (adenomas) and invasive stages point towards a more intricate role of the SHP-2 protein in tumorigenesis. Adenomas and early invasive (stage I) lesions showed higher SHP-2 expression than more advanced tumors [50]. In addition to gastrointestinal malignancies, SHP-2 has been studied in thyroid, oral and laryngeal, and prostate and gynecological system neoplasms, with heterogeneous and sometimes contradictory results [46]. In thyroid carcinomas and oral and laryngeal squamous cell tumors, SHP-2 demonstrated a similar profile as in the lung, pancreas and stomach, with increased expression in tumor cells compared to non-neoplastic tissue. Moreover, it showcased a positive correlation with advanced tumor stage [51,52,53]. In prostate cancer, conflicting data have been derived with one study demonstrating SHP-2 overexpression, while another one revealed that reduced cytoplasmic staining is associated with tumor development and progression [54,55]. The heterogenous patterns of SHP-2 expression observed in different tumor subtypes clearly showcase the context-dependent role it exerts in tumorigenesis.

## 5. Conclusions

The existing data are mainly preliminary in order to justify an immediate incorporation of SHP-2 targeting in routine treatment protocols of NSCLC. However, our study provides the first analysis of its expression pattern in such an extended cohort of NSCLC cases, giving some initial insight on its potential role as a prognostic and/or predictive biomarker. Based on our findings, it could serve as a very reliable predictor of response to ICIs, even though additional studies are required in order to validate our results.

## Figures and Tables

**Table 1:** Epidemiological and clinical characteristics of our study population.

Parameter	Median	Range
Age (years)	66	34–88
Gender	Number	Percentage (%)
Male	184	71.3
Female	74	28.7
Smoking		
Yes	200	77.5
No	58	22.5
Histological subtype		
Adenocarcinoma	219	84.9
Squamous cell carcinoma	28	10.9
Non-small-cell lung cancer	10	3.9
Neuroendocrine tumor	1	0.3
Immunotherapy		
Yes	136	52.7
No	122	47.3
Survival (months)	Median	Range
Overall Survival (OS)	11	1–50
Progression-Free Survival (PFS)	3	0–36

**Table 2:** Molecular alterations of our specimens.

Genetic Alteration	Number	Percentage (%)
*EGFR* mutation		
Yes	24	9.3
Ex19del	15	5.8
Ex20 (L858R)	5	1.9
Ex21ins	4	1.6
No	234	90.7
*KRAS* mutation		
Yes	70	27.1
No	188	72.9
*BRAF* mutation		
Yes	5	1.9
No	253	98.1
*ALK* amplification		
Yes	9	3.5
No	249	96.5

**Table 3:** ROS1 and SHP-2 immunohistochemical expression.

Protein	Number of Positive Cases (% Percentage)
ROS1	21
Adenocarcinoma	20 (9.1%)
Squamous cell carcinoma	0
Non-small-cell lung cancer-NOS	1 (10%)
Neuroendocrine tumor	0
SHP-2	60
Adenocarcinoma	49
Squamous cell carcinoma	9
Non-small-cell lung cancer-NOS	2 (20%)
Neuroendocrine tumor	0

## Data Availability

The original contributions presented in this study are included in the article. Further inquiries can be directed to the corresponding author.

## Abbreviations

The following abbreviations are used in this manuscript:

NSCLC	Non-small-cell lung cancer
SCLC	Small-cell lung cancer
PFS	Progression-free survival
OS	Overall survival
NOS	Not otherwise specified
ICIs	Immune checkpoint inhibitors
NCCN	National Comprehensive Cancer Network
NKUA	National and Kapodistrian University of Athens
TCGA	The Cancer Genome Atlas
RTK	Tyrosine Kinase Receptor
CCSP	Clara cell secretory protein
rtTA	Reverse tetracycline transactivator
EMT	Epithelial-to-mesenchymal transition
3-AG	3-acetoxylteuvincenone G
Cas9	CRISPR-associated protein 9
